# Developing female optometrists while challenging gender norms

**Published:** 2025-03-07

**Authors:** Mritunjay Tiwary, Kriti Shukla

**Affiliations:** 1 CEO and Executive Trustee: Akhand Jyoti Eye Hospitals, India.; 2 Editor: *Community Eye Health Journal* – South Asia Edition, India.

**Girls in rural Bihar are empowered through football and optometry training.**

**Figure:**
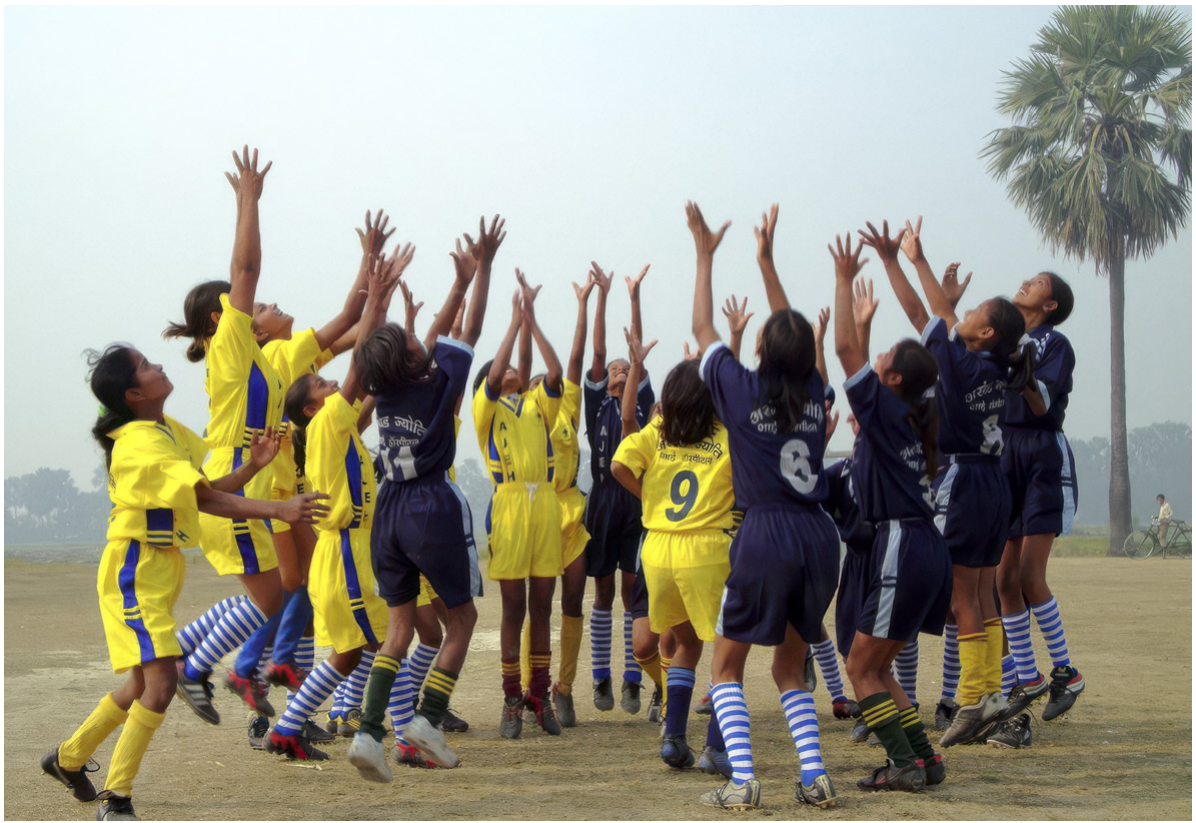
The Akhand Jyoti football team. india

© MRITUNJAY TIWARY CC BY-NC-SA 4.0

The Football to Eyeball Girls Education Programme, a sports-based education and career-development programme, was launched in 2009 by Akhand Jyoti, a non-profit organisation in Mastichak in the state of Bihar, for schoolgirls from rural, economically disadvantaged backgrounds.

The inspiration behind the programme was an incident involving a 15-year-old girl in rural Bihar who was sold by her parents to work as a domestic labourer.

The programme was initiated as a way to build confidence in girls through football, a sport traditionally played by boys and men, and to challenge stereotypical gender norms. It has now grown into a structured initiative that trains young girls in football and in optometry, equipping them with skills for a career in either the eye health sector or professional football.

Girls aged 12–16 years are recruited into the sport and study programme through a network of 44 vision centres and 1,500 outreach volunteers spread across Bihar. The volunteers assess the financial stiuation of the applicant's family, and give priority to girls from financially disadvantaged backgrounds. The parents of enrolled girls subsequently act as ‘brand ambassadors’, helping to promote the programme.

“The programme has now grown into a structured initiative that trains young girls football and in optometry and eye care.”

There is an entrance examination for applicants, to test their proficiency in primary-level English language and O-level science, followed by a personal interview with both the applicant and her parents. Currently, the programme receives around 1,000 applications, from which cohorts of 150 to 200 applicants are selected. All girls are taught life skills in the foundation year. Thereafter, they are trained in their chosen careers: optometry (roughly two-thirds of the cohort) or professional football (with the aim of securing a place in state and national teams). They can also choose to pursue a dual role – as an optometrist and as a footballer. Students have the opportunity to pursue a business management course after completing the bachelor's degree in optometry.

The Football to Eyeball Girls Education Programme has transformed the lives of the girls involved, their families, and the wider community – both socially and economically. They have pursued higher education, secured stable employment, and even taken on leadership roles. Currently, graduates from this programme account for more than 60% of all leadership positions at Akhand Jyoti Eye Hospitals.

Graduates are employed as qualified optometrists in Akhand Jyoti Eye Hospitals or elsewhere, earning between Rs 20,000 (roughly US $230) and Rs 40,000 (roughly US $460) per month. Delaying the age of marriage and motherhood has improved their health and well-being, and they are inspiring other girls to follow a similar path. Importantly, men in the community have begun recognising the value of education and financial independence for girls.

## Challenges

There was significant initial resistance to the programme in the deeply patriarchal environment of rural Bihar. Families were unwilling to let their daughters step outside their homes, let alone play football. Gradually, mindsets changed, as the tangible benefits of education for girls, their financial independence, and secure employment became evident. Success stories, such as that of Manishe Dwivedi, have inspired more families to support their daughters’ education and career aspirations. Hailing from a low-income, disadvantaged family in Siwan, Bihar, Manisha joined the programme at the age of 14. She completed her bachelor's degree in optometry, followed by a degree in management. Today, she leads the Football to Eyeball Girls Education Programme, mentoring young girls to follow in her footsteps.

To date, 725 girls have been enrolled in the programme. A total of 485 have been trained as optometrists, and 15 have successfully qualified to play football for the Indian national and Bihar State Teams. The programme aims to empower 1,500 girls by 2030. By extending this innovative model to other underserved regions, it aims to replicate the model of sports, education, and employment to bring about gender equality and social change.

